# Discrete particle model for cement infiltration within open-cell structures: Prevention of osteoporotic fracture

**DOI:** 10.1371/journal.pone.0199035

**Published:** 2018-06-13

**Authors:** Samuel Jesús Ramos-Infante, Amadeo Ten-Esteve, Angel Alberich-Bayarri, María Angeles Pérez

**Affiliations:** 1 M2BE-Multiscale in Mechanical and Biological Engineering, Departamento de Ingeniería Mecánica, Instituto de Investigación Sanitaria Aragón (IIS Aragón), Instituto de Investigación en Ingeniería de Aragón (I3A), Universidad de Zaragoza, Zaragoza, Spain; 2 GIBI230, Biomedical Imaging Research Group, La Fe Health Research Institute, Valencia, Spain; 3 QUIBIM SL, Quantitative Imaging Biomarkers in Medicine, Valencia, Spain; Klinikum rechts der Isar der Technischen Universitat Munchen, GERMANY

## Abstract

This paper proposes a discrete particle model based on the random-walk theory for simulating cement infiltration within open-cell structures to prevent osteoporotic proximal femur fractures. Model parameters consider the cement viscosity (high and low) and the desired direction of injection (vertical and diagonal). *In vitro* and *in silico* characterizations of augmented open-cell structures validated the computational model and quantified the improved mechanical properties (Young’s modulus) of the augmented specimens.

The cement injection pattern was successfully predicted in all the simulated cases. All the augmented specimens exhibited enhanced mechanical properties computationally and experimentally (maximum improvements of 237.95 ± 12.91% and 246.85 ± 35.57%, respectively). The open-cell structures with high porosity fraction showed a considerable increase in mechanical properties. Cement augmentation in low porosity fraction specimens resulted in a lesser increase in mechanical properties. The results suggest that the proposed discrete particle model is adequate for use as a femoroplasty planning framework.

## 1. Introduction

Osteoporosis is a skeletal disease characterized by low bone mineral density (BMD) and micro-architectural deterioration of bone tissue, leading to increased bone fragility and risk of fracture [1]. Osteoporotic proximal femur fractures are associated with high morbidity and dramatically reduce a patient´s quality of life [2]. Although these events account for less than 20% of all osteoporotic fractures, they represent the majority of fracture-related health care expenditure and mortality in men and women over the age of 50 years [3].

Current preventive measures include lifestyle interventions, fall prevention and hip protectors [4–6]. A variety of drugs have been tested but are limited in efficacy due to long delays in restoring bone strength, high costs, and side-effects such as an increased risk of cancer [7–9]. Because morbidity associated with such fractures has a significant socioeconomic cost [10], various treatments have been proposed to increase bone mass and decrease fracture incidence. One such treatment is the mechanical reinforcement of functionally relevant osteoporotic bones such as the femur [11]. Femoroplasty is the process of injecting polymethylmethacrylate (PMMA) hereafter referred to as cement, into the proximal femur to prevent osteoporotic hip fracture [12–14]. Femoroplasty increases the strength and energy to failure of the femur and can be performed in a minimally-invasively manner with lower hospitalization costs and reduced recovery time [12,15]. The reinforcement is achieved via percutaneous cement injection to prevent progressive deformity or collapse and to alleviate disabling pain [16]. Initially, the injected material takes the form of a viscous dough, and a few minutes after injection into the bone, the dough polymerizes and solidifies.

A vast number of published studies [12,13,16–21] have concluded that after augmentation using cement, osteoporotic femurs may become significantly stronger, offering a reduced risk of fracture [13,14,22]. First-generation femoroplasty approaches resulted in significant improvements in both fracture load and energy compared with those on the non-augmented contralateral side [11,13,14]. However, an elevated risk of biological impairment was recognized due to heat, toxicity, pressure, leakage or blockage of the blood support associated with the large cement volume. Therefore, in second-generation femoroplasty studies, the amount of cement was decreased [12,15,23,24]. Additionally, suboptimal injection can result in bone weakening due to stress concentration, primarily at the cement-bone interface, rendering the augmentation unsuccessful [25].

Another study revealed that the location of the cement cloud influences the biomechanical outcome [12]. However, further investigations are currently seeking to identify the ideal augmentation strategy [26]. Customized treatments require special planning and controlled injection techniques that are not widely available. The goal can be stated as an optimization problem, the solution of which is sought through the application of a robust optimization procedure. Until now, notably few papers have been published in this direction. A variation of the well-known bidirectional evolutionary structural optimization (BESO) method [27] was applied to find the minimum volume of cement needed to increase the predicted yield load of the specimens [25] and to optimize the cement pattern for femoroplasty [28]. Additionally, computational fluid dynamics (CFD) simulations of cement injection into the trabecular structure have been performed to investigate the treatment and impact of injected cement [29]. In addition, a deterministic method based on sequential quadratic programming (SQP) was completed to evaluate the influence of certain parameters on the cement distribution [10]. Although new evolutionary optimization methods for the augmentation of osteoporotic bones have been developed, none have been validated with experimental studies [10,25,26,28,29]. With respect to experimental validation, the method of smoothed particle hydrodynamics (SPH) has been utilized to model the flow of cement inside porous media with the incorporation of different viscosities [30]. Although certain studies qualitatively compared three-dimensional results with those obtained in experiments, only the cement cloud [30] and bone infiltration [31] inside trabecular bone were studied. Therefore, mechanical property improvements were not assessed computationally or experimentally.

According to the literature, the best augmentation strategy is currently unknown [28]. Due to high computational costs, particle models have gained popularity for modelling fluid flows [32]. Therefore, the main goal of this work is the development of a discrete particle model for cement infiltration.

We performed an *in vitro* and *in silico* characterization of augmented open-cell structures to assess qualitative and quantitative results. The infiltration of two commercial cement types with different viscosities (high- and low-viscosity) within open-cell structures (Sawbones; Malmö, Sweden) of three different porosities was analysed. To validate the proposed model, *in vitro* experiments were performed, and the results were compared with *in silico* finite element (FE) simulations. We demonstrate that cement injection increases the mechanical properties (Young’s modulus) of open-cell structures resembling different trabecular bone structures. Furthermore, cement viscosity affects the mechanical performance of the augmented open-cell structures. The main novelties of this work are the proposed *in vitro* experiments used to validate the *in silico* approach and the employment of two cement viscosities and three open-cell structures with different porosity fractions.

## 2. Materials and methods

**Fig 1:**
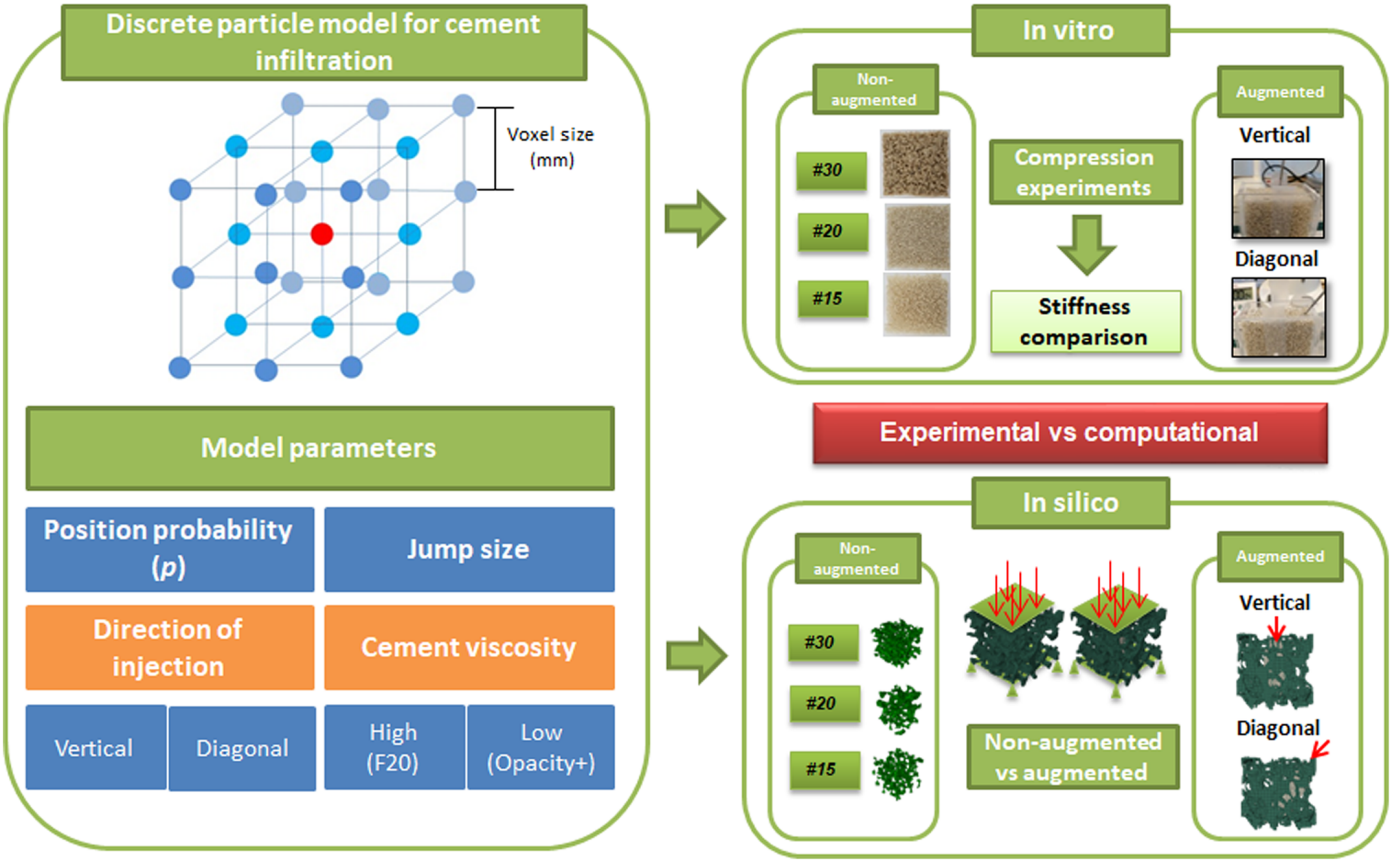
Workflow for the in vitro and in silico characterization of open-cell structures: Non-augmented vs. augmented with cement.

A discrete particle model for cement infiltration based on the random-walk theory [33] is presented in this section (Fig 1), and *in vitro* and *in silico* characterizations of augmented open-cell structures are described (Fig 1). *In vitro* and *in silico* characterizations of non-augmented open-cell structures were performed in a previous study [34].

### 2.1 Discrete particle model for cement infiltration

**Fig 2:**
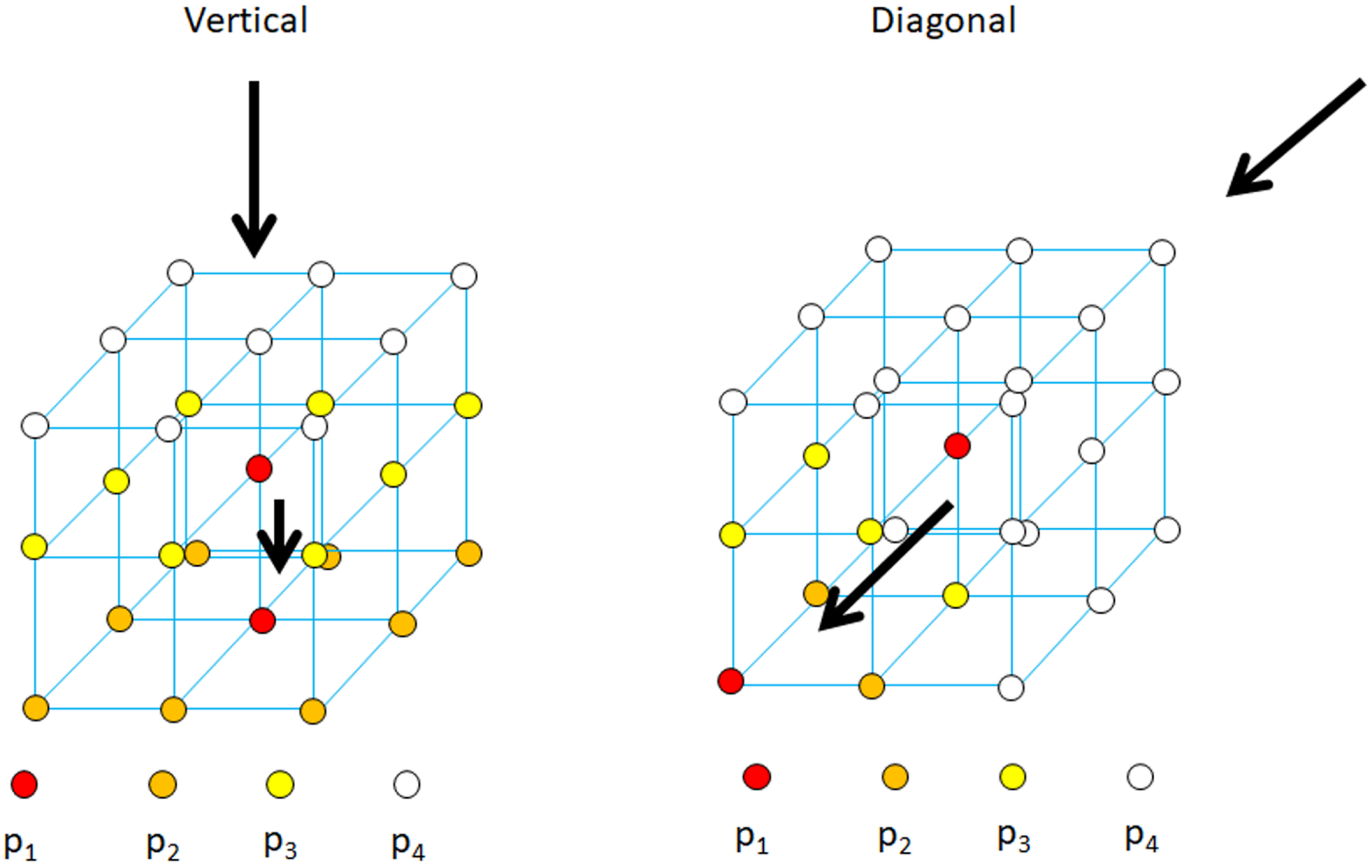
Probabilities depending on the desired direction of injection.

The complexity of *in vitro* testing led to the planning and computational simulation of cement infiltration through a porous medium resembling the trabecular bone structure. An approach for modelling the cement infiltration based on the random-walk theory [33] was proposed. This phenomenological model allowed us to control selected parameters (viscosity and direction of injection) that are important in planning the femoroplasty technique. Initially, a cement particle is assumed to be surrounded by 26 locations that could be occupied by a particle (Figs 1 and 2). The cement particle distance depends on the voxel size (Section 2.3). Cement particles are not allowed to remain in their initial position. Therefore, a cement particle is moved to another controlled location. We opted for an anisotropic diffusion, i.e., cement particles can occupy neighbouring positions with different probabilities *p* depending on the desired direction of injection (Fig 2). We considered two directions of injection: vertical and diagonal. In each case, the neighbouring cement particle positions are evaluated, and depending on the available states, the corresponding value of *p* is computed to fulfil $\sum_{i\;=\;1}^{n1}p_{1}+\sum_{i\;=\;1}^{n2}p_{2}+\sum_{i\;=\;1}^{n3}p_{3}+\sum_{i\;=\;1}^{n4}p_{4}\;=\;1$ (Fig 2). For the vertical direction of injection, a strongly preferred upright direction was assumed as *p*_1_ = 15*p*_2_ = 50*p*_3_ = 90*p*_4_, and in this case, *p* can be calculated as $\frac{150}{269}$. For the diagonal direction of injection, the oblique direction is the preferred direction, which was assumed as *p*_1_ = 5*p*_2_ = 20*p*_3_ = 90*p*_4_ with *p* equal to $\frac{90}{163}$. Additionally, the model incorporated “contact inhibition” by searching for vacant positions when a cement particle moves, depending on the available positions. The model considers that the positions representing the bone trabeculae cannot be occupied by cement particles. At the end of the injection, the availability of the final position is verified. If that position is not free (bone or cement particle position), another neighbouring location is randomly chosen. The cement viscosity was considered in our model as the jump size that a particle could undergo in each iteration (Fig 1). Basically, this jump size represents the shear rate for a constant shear stress. Therefore, the jump size that a particle could undergo in each iteration increased as the cement viscosity decreased. For high-viscosity cement, the jump size was assumed as one voxel, whereas for low-viscosity, the jump size was equal to five voxels. This parameter takes into account in a phenomenological manner, the different diffusive capacity due to cement viscosity. Notably, a cement particle finds more free positions as the infiltration increases, i.e., as the cement viscosity decreases.

The number of cement particles injected (N_injected particles_) depends on the cement volume injected (V_cement_) and the cement particle volume (V_cement particle_), which is directly related to the voxel volume (Section 2.3) as V_cement_ = N_injected particles_ * V_cement particle_.

### 2.2 *In vitro* characterization of augmented open-cell structures

The augmentation of three open-cell structures (Sawbones; Malmö, Sweden) was studied with three different densities similar to that of trabecular bone (Table 1). Hereafter, we refer to these items as specimen #15 (Sawbones; product no. 1522-526-1; Malmö, Sweden), specimen #20 (Sawbones; product no. 1522–524; Malmö, Sweden) and specimen #30 (Sawbones; product no. 1522–525; Malmö, Sweden). The *in vitro* characterization of non-augmented open-cell structures was previously conducted (34) (Table 1). Twelve open-cell structures (#15, #20, #30) were cut into blocks of approximately 65 x 65 x 40 mm. Each block was enclosed in a Plexiglass shell of 5 mm thickness, as the cortical shell. Two different commercial cements were injected, i.e., F20 (Teknimed, Toulouse, France) and Opacity+ cement (Teknimed), which have high- and low-viscosity, respectively. A commercial cement injection system (Teknimed S5Kit; Teknimed S.A.S, France) was used in the cement augmentation procedure. The corresponding cement instructions for mixing were followed. Four millilitres of cement was injected into each specimen through a drilled hole 3 mm in diameter on the top face (vertical direction) (Fig 1), but the effect of injection in a diagonal direction was also analysed through a 3 mm hole on the corner top face (Fig 1). Two open-cell structures of each density were analysed in each direction (vertical and diagonal) with the two cement types (Fig 1). The injection procedure was repeated for all the prepared specimens. After 24 h of cement curing, compression mechanical tests were performed using a servo-hydraulic material testing machine (Microtest; model EFH, Spain). Each specimen was placed between steel plates at room temperature (approx. 23 °C) and loaded in the direction of the axis of symmetry. The quasi-static compression load was measured with a commercial load cell (10 kN) applied at a constant velocity rate of 1 mm/min [34]. From the force-displacement curve, Young’s modulus of each specimen was estimated, and the increase in mechanical properties was calculated.

**Table 1:** Open-cell specimens, densities, porosities, mean experimental Young’s modulus [34] and the mean computational Young’s modulus [34].

Specimen	Number of specimens	Density (g/cc)	Porosity specifications (%)	Experimental E (34) (MPa)	Computational E (34) (MPa)
**#15**	4	0.24	85	62.74 ± 4.14	85.89 ± 22.33
**#20**	4	0.32	79	111.35 ± 8.24	121.16 ± 27.36
**#30**	4	0.48	69	187.47 ± 20.53	178.05 ± 39.44

### 2.3 *In silico* characterization of augmented open-cell structures

*In silico* characterization of non-augmented open-cell structures was previously conducted [34]. The obtained mean results are shown in Table 1, and the process is revised in this work. Prior to cement augmentation of the open-cell structures, computed tomography (CT) acquisition was performed in a Phillips Brilliance system using 64 detectors with the following parameters: slice thickness = 0.672 mm, KVP = 120, spacing between slices = 0.672 mm and pixel spacing = 0.234 mm. A 3D bicubic interpolation algorithm was applied to reduce the slice thickness to 0.16 mm (voxel size). The interpolated images were reconstructed using a semiautomatic reconstruction (MIMICS, Materialise NV; Leuven, Belgium). The specimens were digitally cut, and a representative volume element was chosen [34]. A voxel mesh was generated using the voxel create mesh module (MIMICS, Materialise NV; Leuven, Belgium).

Therefore, the discrete particle model for cement infiltration (Section 2.1) was run within the voxel mesh created for each specimen. As indicated, two directions of injection and two cement viscosities were modelled. Once the cement injection was simulated, a new voxel mesh was generated (bone plus cement) to simulate the compression test. The boundary condition for the voxel mesh was based on an idealization of those in a uniaxial compression test [35]. A uniaxial displacement (strain of 2%) was applied to the top surface of the cubic bone samples, and the bottom surface was kept fixed [35].

The bone and cement were assumed to be linear elastic and isotropic with Young’s modulus of 3200 MPa (Sawbones; Malmö, Sweden) and 2000 MPa [36], respectively. Poisson´s ratio was defined as 0.3.

Non-linear FE analyses were performed in ABAQUS v6.14 (Dassault Systèmes Simulia Corp., Suresnes Frances) and run in a computational cluster of 224 cores with 576 GB of RAM. After FE analysis, the augmented mechanical properties (Young’s modulus) were estimated to calculate the final improvement of the specimen mechanical properties. Prior to the experimental compression tests, CT acquisition of the augmented specimens was again performed (one acquisition per cement type, direction of injection and open-cell structure type). In this case, the cement clouds and filling patterns inside the open-cell structures were reconstructed [30], and their sphericity was calculated [37]. Statistical analysis of the results was performed. A dependent samples t test was applied to determine whether statistically significant differences were identified. Additionally, Pearson correlation coefficients were calculated.

## 3. Results

**Fig 3:**
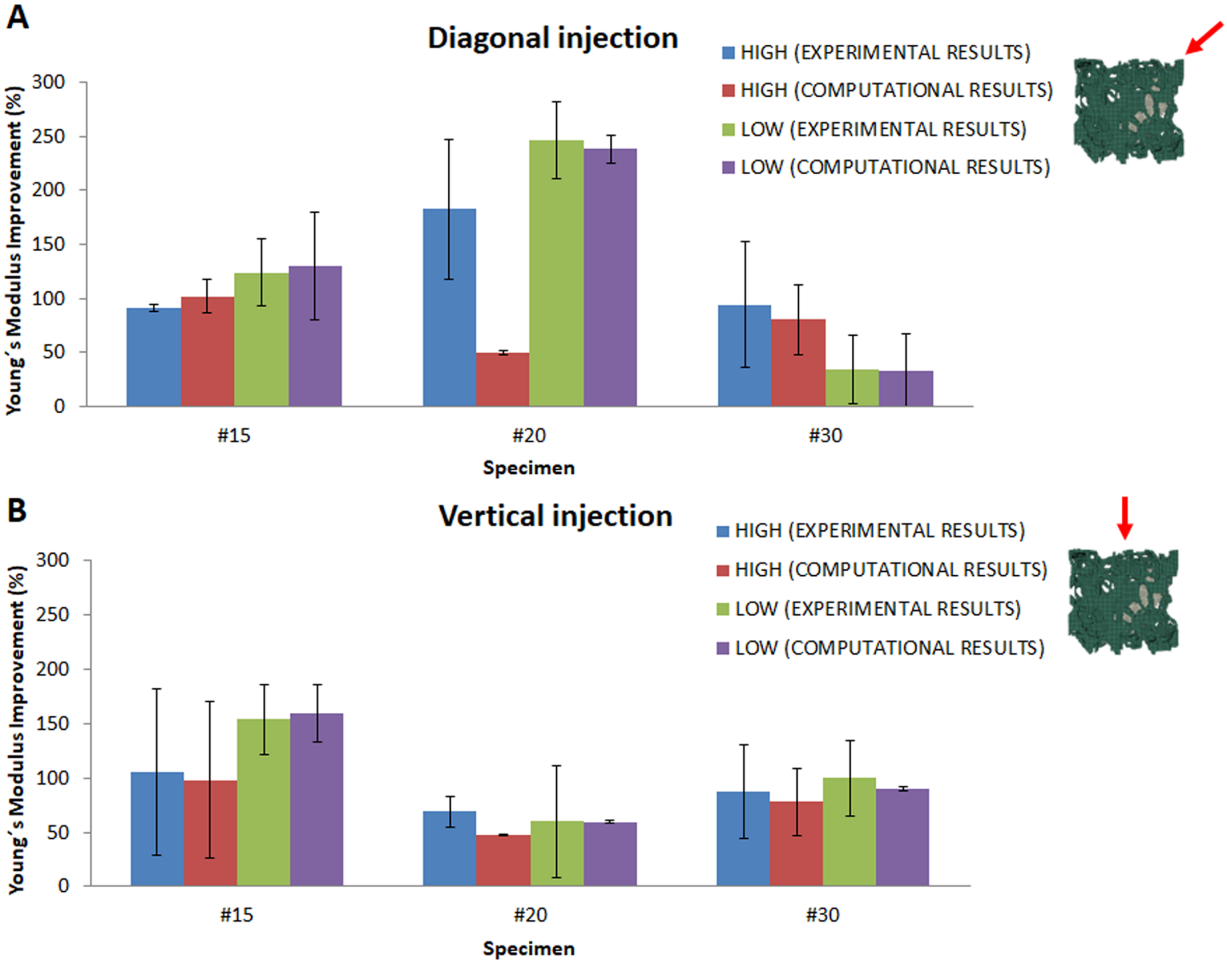
Mean Young’s modulus improvement (%) in all the cases tested in vitro and in silico: (A) vertical and (B) diagonal directions of injection. Bars indicate the standard deviation values (see Table 2).

In general, the augmented specimens exhibited enhanced mechanical properties regardless of the direction of injection, cement viscosity or open-cell structure type (Table 2 and Fig 3). Low-viscosity cement showed better improvements for all the specimens and directions, except for specimen #30 and the diagonal direction (see Table 2 and Fig 3B). As specimen #30 showed the lowest porosity fraction (see Table 1), both cement viscosities were difficult to inject using the commercial injection system because cement was not able to reach neighbour pores as easily as it was in specimens #15 and #20, which had high porosity fractions. Although all the augmented specimens exhibited increased mechanical properties for all cement viscosities, the specimen with the highest porosity fraction (specimen #15), similar to osteoporotic bone, showed considerable improvements in mechanical properties (Fig 3) because the cement was able to infiltrate more fully. In addition, similar mechanical property improvements were achieved regardless of the direction of injection. However, we noted certain differences in specimen #20 (Fig 3), for which the diagonal injection showed better Young’s modulus improvements.

**Table 2:** Mean Young’s modulus improvement (%) in all the cases tested in vitro and in silico. STD indicates standard deviation. Bold numbers in the p-value column indicated a negative (-1) Pearson correlation coefficient.

		SPECIMEN	*IN VITRO* IMPROVEMENT (%)	MEAN (STD) *IN VITRO* IMPROVEMENT (%)	*IN SILICO* IMPROVEMENT (%)	MEAN (STD) *IN SILICO* IMPROVEMENT (%)	P-VALUE (T-STUDENT)
**HIGH**	**DIAGONAL**	HIGH_15_1	87.4	91.0 (3.7)	86.3	101.9 (15.6)	0.53
		HIGH_15_2	94.7		117.5		
	**VERTICAL**	HIGH_15_3	29.1	105.7 (76.6)	26.5	98.33 (71.79)	0.37
		HIGH_15_4	182.3		170.1		
	**DIAGONAL**	HIGH_20_1	118.1	182.8 (64.7)	51.4	49.31 (2.07)	**0.29**
		HIGH_20_2	247.4		47.2		
	**VERTICAL**	HIGH_20_3	54.7	68.9 (14.2)	47.8	47.62 (0.19)	**0.38**
		HIGH_20_4	83.2		47.4		
	**DIAGONAL**	HIGH_30_1	35.5	93.9 (58.4)	47.9	80.46 (32.6)	0.69
		HIGH_30_2	152.4		113.1		
	**VERTICAL**	HIGH_30_3	44.8	87.8 (43.0)	47.2	77.98 (30.74)	0.57
		HIGH_30_4	130.7		108.7		
**LOW**	**DIAGONAL**	LOW_15_1	93.1	123.9 (30.7)	80.6	130.01 (49.44)	0.80
		LOW_15_2	154.6		179.5		
	**VERTICAL**	LOW_15_3	186.4	154.3 (32.1)	186.0	159.29 (26.73)	0.52
		LOW_15_4	122.1		132.6		
	**DIAGONAL**	LOW_20_1	211.3	246.9 (35.6)	225.0	237.95 (12.91)	0.76
		LOW_20_2	282.4		250.9		
	**VERTICAL**	LOW_20_3	111.7	60.1 (51.7)	58.3	59.74 (1.47)	**0.99**
		LOW_20_4	8.4		61.2		
	**DIAGONAL**	LOW_30_1	66.3	34.1 (32.1)	66.7	33.38 (33.28)	0.63
		LOW_30_2	2.0		0.1		
	**VERTICAL**	LOW_30_3	64.9	99.9 (35.0)	88.7	90.21 (1.47)	0.82
		LOW_30_4	135.0		91.7		

The computational predictions were notably close to the experimental values (see Table 2 and Fig 3). None of the results presented statistically significant differences between the computational and experimental results (p>0.05, t-student). The computational results for specimen #20 with high-viscosity cement (vertical and diagonal) and low-viscosity cement (only vertical) compared poorly with the experimental results (the Pearson correlation coefficient was -1, see bold numbers in Table 2).

**Fig 4:**
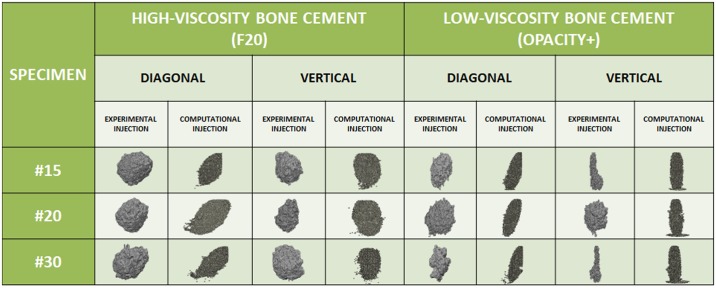
Qualitative comparison of the cement infiltration patterns within certain open-cell structures in each case simulated.

To further validate the model, the filling pattern was successfully predicted based on a comparison of the computational and experimental infiltration (Fig 4). High-viscosity cement created a denser cement volume, whereas low-viscosity cement tended to spread more fully inside the trabecular bone. The sphericity of the injected cement was quantified in Table 3. The sphericity was higher with high-viscosity cement than with low-viscosity cement. Most of the results did not presented statistical significant differences between the experimental and computational results (p>0.05, t-student). Only when low-viscosity cement was injected in the vertical direction were significant differences observed for specimens #15 and #20 (see Table 3, last column numbers in italics). The Pearson correlation coefficient was positive (= 1) in all cases.

**Table 3:** Sphericity of the cement cloud in all cases tested in vitro and in silico. STD indicates standard deviation.

		SPECIMEN	*IN VITRO* SPHERICITY	MEAN *IN VITRO* (STD) SPHERICITY	IN *SILICO* SPHERICITY	MEAN (STD) *IN SILICO* SPHERICITY	P-VALUE (T-STUDENT)
**HIGH**	**DIAGONAL**	HIGH_15_1	0.72	0.80 (0.07)	0.81	0.82 (0.02)	0.74
		HIGH_15_2	0.87		0.84		
	**VERTICAL**	HIGH_15_3	0.71	0.74 (0.04)	0.86	0.87 (0.01)	0.15
		HIGH_15_4	0.78		0.88		
	**DIAGONAL**	HIGH_20_1	0.93	0.86 (0.06)	0.79	0.77 (0.02)	0.30
		HIGH_20_2	0.80		0.75		
	**VERTICAL**	HIGH_20_3	0.84	0.82 (0.01)	0.86	0.83 (0.03)	0.66
		HIGH_20_4	0.81		0.80		
	**DIAGONAL**	HIGH_30_1	0.73	0.80 (0.07)	0.73	0.75 (0.02)	0.46
		HIGH_30_2	0.86		0.77		
	**VERTICAL**	HIGH_30_3	0.86	0.87 (0.004)	0.80	0.73 (0.02)	0.26
		HIGH_30_4	0.87		0.85		
**LOW**	**DIAGONAL**	LOW_15_1	0.74	0.59 (0.15)	0.53	0.52 (0.005)	0.73
		LOW_15_2	0.44		0.52		
	**VERTICAL**	LOW_15_3	0.80	0.82 (0.02)	0.55	0.56 (0.01)	*0*.*03*
		LOW_15_4	0.84		0.57		
	**DIAGONAL**	LOW_20_1	0.79	0.71 (0.08)	0.53	0.53 (0.03)	0.22
		LOW_20_2	0.64		0.52		
	**VERTICAL**	LOW_20_3	0.78	0.74 (0.04)	0.59	0.56 (0.03)	*0*.*03*
		LOW_20_4	0.70		0.53		
	**DIAGONAL**	LOW_30_1	0.63	0.51 (0.12)	0.57	0.55 (0.02)	0.75
		LOW_30_2	0.39		0.53		
	**VERTICAL**	LOW_30_3	0.68	0.74 (0.06)	0.56	0.57 (0.01)	0.18
		LOW_30_4	0.80		0.58		

## 4. Discussion

Augmentation of an osteoporotic femur using cement to prevent or reduce the risk of fracture has been suggested as an alternative preventive treatment [30]. The results of the current study support our original hypothesis that femoroplasty increases the mechanical properties compared with non-augmented controls (Table 2 and Fig 3). A few recent studies have reported attempts at restoring the mechanical strength of femur specimens using a relatively small amount of infiltrated cement with limited or no success [12,15,18,24]. The procedure requires precise planning and execution. Effective planning relies on (among other factors) an accurate method for predicting the diffusion of the cement through the porous medium of osteoporotic trabecular bone. A crucial step in the planning process is to determine the optimum volume and filling pattern of the cement such that the best outcome is achieved [30]. A successful planning framework should include a module for predicting the cement infiltration inside trabecular bone. The majority of fragility fractures occur at trabecular-dominant bone sites. Indeed, the trabecular bone plays important roles in the load transmission and energy absorption in major joints.

Our goal was to develop a discrete particle model for cement infiltration based on the random-walk theory [33]. Random-walk on a grid is similar to methods used in lattice gas and Lattice Boltzmann simulations of diffusion without convection [38]. The main novelty of the current work is that the proposed model was qualitatively and quantitatively validated through *in vitro* experiments using two cement viscosities and three different open-cell structures.

We performed an experimental set of validation tests using non-augmented specimens as surrogate trabecular bone tissue and injected 4 ml of cement in a controlled manner. This amount is far less than the amounts used in first-generation femoroplasty experiments [11,13,14], in which approximately 40–50 ml of cement was needed to obtain a 30–40% increase in the fracture load. Second-generation femoroplasty approaches resulted in mechanical property improvements of more than 100% when 12 ml of cement was infiltrated [25], even though the model was not experimentally validated. Finally, a recent study achieved an increase in mechanical properties of more than 100% by injecting approximately 10 ml of cement [26]. All previous studies agree in augmenting the upper side of the femoral neck, where the maximum traction loads are reached. In fact, augmentation of the superior and inferior position of the femoral neck close to the cortex results in the most favourable outcome [39]. This observation supports the hypothesis that the use of subject-specific models and optimization, combined with intra-operative tools for precise cement delivery, reduces the required cement volume [26].

Two cement viscosities were used in this work, and the simulation and experimental results were compared. Strong correlations between experimental and simulation results were obtained for spreading distance and cement clouds (Fig 4). The cement pattern created inside the open-cell structures by the discrete particle model involved augmentation following the vertical and diagonal directions, similar to the directions inside the femoral neck. The material distribution was highly similar to the results obtained in the literature [25,30]. Our model showed that 4 ml of cement resulted in Young’s modulus increases ranging from 91.04% (high-viscosity cement) to 154.29% (low-viscosity cement) in specimen #15 (Fig 3), which had a porosity fraction close to that of the osteoporotic femur. The target Young’s modulus in the current work was set to nearly 20% higher than Young’s modulus of a healthy trabecular femur (_Ehealthy trabecular femur_ ~ 11.4 GPa) [40], although the proposed model supplies sufficient versatility to set the target to any desired value depending on the direction of injection and cement viscosity. Notably, the infiltration of the two cements showed different results depending on the direction of injection and cement viscosity. In most cases, excellent agreement between the experimental and computational results was achieved and there were no statistically significant differences between the two results (Fig 3 and Table 2). Specimen #20 showed a particular increase in mechanical properties when cement was infiltrated in the diagonal direction with respect to the other specimens (Fig 3B). In contrast, when cement was infiltrated in the vertical direction, the improvement in mechanical properties was lower (Fig 3A). However, when performing statistical analysis between both directions of injection, no statistically significant differences were estimated (p = 0.26, t-student for high-viscosity cement, p = 0.27, t-student for low-viscosity cement). In addition, for the diagonal direction, important differences between the computational and experimental results were observed for high-viscosity cement in specimen #20 (Fig 3). There could be two reasons for these differences. First, the manufacturing process of the open-cell structures could lead to a decrease in porosity and a change in the micro-architecture of the specimen itself. Second, the position of the structure formed by the solidified cement within the open-cell structure could affect the final mechanical properties. For example, if the solidified structure happens to form at the weak-point of the open-cell structure, a more important enhancement of the mechanical properties could result. Therefore, we cannot conclude that cement diffusion is the only crucial mechanism in the improvement of mechanical properties; the direction of injection, the specimen manufacturing process and its micro-architecture and the final position of the structured-formed must also be considered. Human trabecular bone is anisotropic by nature. Additionally, the cement viscosity affected the compactness of the cement final shape. A high-viscosity cement produces a cement cloud with high sphericity (Table 3 and Fig 4). This observation suggests that medium or low cement viscosities (low sphericity) are ideal for injections inside porous media, including osteoporotic trabecular bone, because the final shape is sufficiently compact [30].

Notably, the proposed model was used in conjunction with FE analyses to predict the effect of various hypothetical augmentation scenarios on the mechanical properties of bone [30]. An increase in the mechanical properties was observed regardless of the cement viscosity. In addition, low-viscosity cement showed better Young’s modulus improvements. However, mechanical property improvements were highly similar in specimens #15 and #30, regardless of the direction of injection (Fig 3).

Nevertheless, the proposed methodology presents certain limitations. The validation was performed with only two specimens of each type; therefore, additional data are needed to further validate the model. The probability values assumed for the application of the random-walk theory [33] are mainly phenomenological. Another assumption was the number of voxels considered for the jump size in the low- and high-viscosity cements, considering that more than five voxels (high) generated an unrealistic cement cloud pattern (data are not shown). No previous measurements were collected. However, we have based our hypothesis in experimental data collected from the literature [41]. It is a fact that, considering a simple shear flow, Newton´s law of viscosity relates shear stress, σ, to the velocity gradient or shear rate, γ, through the equation: σ = μγ, where μ is the coefficient of viscosity, or simply the viscosity. For Newtonian fluids, the viscosity is a constant independent of shear rate. However, many fluids, including many polymer solutions and suspensions, are said to be non-Newtonian, and the viscosity is not a coefficient but a function of the shear rate and/or the time of shearing. For example, it is common for viscosity to decrease with increase in shear rate behaviour known as “shear thinning”. Conversely, it is possible for viscosity to increase with shear rate referred to as “shear thickening”. Alternatively, it is possible that at a constant shear stress the viscosity decreases over time. In our particular model, we have hypothesized that the viscosity is a function of the jump size (or shear rate) that a particle could undergo. For a constant value of the shear stress, the viscosity decreases as the jump size increases its value. For instance, for high-viscosity cement, the jump size was assumed to be one voxel, whereas for low-viscosity cement, the jump size was equal to five voxels. This parameter considers, in a phenomenological manner, the different diffusive capacities due to cement viscosity.

With respect to the limitations of the *in silico* characterization, to avoid the long computation times that can arise for more complex analyses [36,42–44], we have used smaller sub-regions to show the correlations between the experimental and computational results [34]. This approximation has resulted in errors as large as 9.5% in predictions of apparent stiffness [42]. However, we obtained similar correlations between the experimental and computational results in non-augmented specimens [34]. Furthermore, injection and pressure rates were not controlled, even though changes in injection rate do not have significant effects on the spread of the cement [45]. In general, small differences were detected between the *in silico* and *in vitro* results. These differences could be due to a loss of accuracy in the image acquisition methodology. The CT images were acquired at the highest in-plane resolution possible, which was limited by the size of the detectors and the field of view of the scanning device. As this CT system is actually used in clinical practice, these conclusions can be translated to obtain similar differences between simulations and real mechanical behaviour. We expect that a finer CT resolution would increase the accuracy of the simulation results, noting that the trabecular structure is very finely spaced, especially for osteoporotic specimens. Nevertheless, increasing the number of voxels also increases the number of fixed particles, which drastically slows the simulations. With the current resolution, our simulations yielded reasonable accuracy, and the added computational cost of finer resolution CT and ionizing radiation dose for patients would not be justified [30]. A change on the voxel size would imply a readjustment of the jump size parameter value. One must consider that the proposed model is intended for use in the preoperative planning of bone augmentation, and computational efficiency is of crucial importance. In our simulations, we ignored the presence of the bone marrow. Selected pilot simulations have demonstrated that considering such a fluid has a negligible effect on the end results [46]. As reasons for this observation, we hypothesize that the bone marrow viscosity is orders of magnitude smaller than the viscosity of the cement [46] and that the interactions between the two fluid particles are minimal. Notably, one of the main problems of the augmentation technique is high temperatures inside the bone during the curing process. Future research must also verify the assumption that by minimizing the injection volume, we can avoid thermal necrosis caused by the exothermic curing process of the cement [28]. Additionally, a validated model for heat generation and propagation could be incorporated into the planning module for the design of safer augmentations by keeping the heat damage away from more vulnerable sites, such as the arteries [47]. Mechanical improvement by means of cement augmentation as reported in the literature does not always translate to zero fracture risk. The risk of fracture also depends on a variety of factors including patient anatomy, height of fall, and floor covering [48–50].

In summary, the cement injection pattern was closely predicted in all the simulated cases (Fig 4 and Table 3), and all the augmented specimens exhibited increased mechanical properties (Fig 3 and Table 2). As the cement injection volume increased, the mechanical properties also improved. In fact, the specimens with the highest porosity fraction (specimen #15) showed a considerable increase in mechanical properties. This increase was mainly due to the high capacity of the cement to diffuse within a more porous trabecular structure.

Therefore, our proposed discrete particle model of cement infiltration allows us to plan and improve cement augmentation in a patient-specific model and also identifies generalizable patterns of cement location that could be applied via simple surgical guidelines. Our model suggests a comprehensive planning strategy that considers several scenarios and can determine the best augmentation strategy for each patient. The results of this study suggest that the chosen method of cement diffusion modelling is an appropriate candidate for our intended application of predicting cement diffusion into the porous structure of trabecular bone.

Femoroplasty significantly increases the mechanical properties when osteoporotic femora are loaded, and cement filling may play an important role in the extent to which femoroplasty affects the mechanical strength of the proximal femur. Consequently, the simplicity and superior performance of the proposed method suggest that it can be used as a tool for optimum subject-specific planning of bone augmentation.
